# Home-monitoring/remote optical coherence tomography in teleophthalmology in patients with eye disorders—a systematic review

**DOI:** 10.3389/fmed.2024.1442758

**Published:** 2024-10-24

**Authors:** Joanna Dolar-Szczasny, Agnieszka Drab, Robert Rejdak

**Affiliations:** ^1^Department of General and Pediatric Ophthalmology, Medical University in Lublin, Lublin, Poland; ^2^Department of Information Technology and Medical Statistics with E-Health Laboratory, Medical University in Lublin, Lublin, Poland

**Keywords:** telemedicine, teleophthalmology, optical coherence tomography, home OCT, remote monitoring

## Abstract

**Introduction:**

Teleophthalmology uses technology to provide remote eye care services, tackling obstacles in accessing specialized care. Optical coherence tomography (OCT) represents a technical advancement, enabling high-resolution ocular imaging. The aim of this study is to evaluate the diagnostic accuracy, feasibility, safety, and clinical utility of home monitoring OCT devices and remote OCT technology compared to standard in-office OCT in teleophthalmology settings across various eye conditions.

**Materials and methods:**

A systematic literature search was conducted in PubMed, Cochrane Library, ScienceDirect and Google Scholar for studies on home-monitoring/remote OCT published from January 2004 to February 2024. Studies utilizing home monitoring/remote OCT in teleophthalmology for patients with eye disorders and reporting on diagnostic accuracy, safety, disease monitoring (clinical utility) or treatment response were included and synthesized narratively.

**Results:**

A total of 12 research studies involving 3,539 participants were incorporated in the analysis. The majority of home or remote OCT scans exhibited satisfactory diagnostic image quality. There was high agreement between home/remote and in-office OCT for detecting pathologies and measuring retinal thickness. Compared to in-person evaluations, home/remote OCT demonstrated excellent sensitivity and specificity, though some variability was seen across conditions and interpreters. Home OCT devices provided feasible and safe self-operation with high patient acceptability. Scan times were faster when conducted at home compared to those in the office.

**Conclusion:**

Home/remote OCT devices can effectively provide diagnostic-grade retinal imaging outside traditional settings. High diagnostic accuracy was demonstrated compared to in-office OCT. Feasibility and patient acceptability data support home OCT for remote monitoring.

## Introduction

Delivering ophthalmic services in the modern healthcare setting is faced with difficulties, such as the requirement for prompt diagnosis and treatment, accessibility issues to specialized care, and the rising incidence of eye disorders (1). The World Health Organization (2) reports that the aging population, the increase in chronic illnesses, and the unequal distribution of healthcare resources around the world have all made these problems worse. Teleophthalmology has surfaced as a viable solution to these problems, utilizing technology developments to offer remote eye care services (3).

According to Rathi et al. (4), teleophthalmology is a paradigm change in eye care that expands the availability of ophthalmic treatments by removing geographical restrictions. Innovative strategies are required to provide complete eye care for everyone due to the global burden of eye illnesses, including glaucoma, diabetic retinopathy, and age-related macular degeneration (AMD) (2). By enabling remote consultations, screenings, and diagnostics, teleophthalmology provides an answer and transforms the field of eye care (5). Particularly in impoverished and rural locations, it has the potential to increase patient outcomes, lower healthcare costs, and improve access to care (6).

Optical coherence tomography (OCT) represents the cutting edge of technical advancement in ophthalmology. Ocular structural cross-sectional imaging at high resolution is made possible by OCT with the use of low-coherence interferometry (7). According to Chopra et al. (7), OCT is a useful tool for the diagnosis and treatment of a variety of ocular disorders due to its non-invasive nature, capacity to record intricate structural information, and real-time imaging capabilities. OCT has been widely utilized to diagnose and track anterior segment abnormalities, glaucoma, and retinal illnesses, offering important insights into the etiology and course of these conditions (8). Conventional OCT equipment, however, has its limits. They are not portable, only available in dedicated rooms within hospital eye clinics and centers, and frequently require pupil dilation, involving trained ophthalmic technicians for their operation which can be not convenient for patients (7). Furthermore, interpreting OCT scans can be difficult and time-consuming, necessitating image analysis experience (9).

In an effort to get beyond these restrictions, OCT technology has recently advanced. Notal Vision Home OCT (NVHO) and other home monitoring OCT devices allow patients to self-administer OCT scans and provide the results to their healthcare practitioners for remote monitoring (10). These gadgets have demonstrated potential in raising patient adherence and assisting in the early identification of illness progression (7). Additionally, developments in remote OCT technology have made it possible to obtain high-quality, real-time imaging from a distance. Because they do not require pupil dilation to obtain high-resolution pictures, handheld, portable OCT devices like the Bioptigen Envisu C-Class are ideal for teleophthalmology applications (8). Furthermore, cloud-based platforms have been created to make it easier for OCT data to be securely transmitted, stored, and analyzed. This allows healthcare providers to collaborate and consult remotely (11).

OCT has a lot of potential for teleophthalmology, but the research that is now available shows a mixed picture with gaps, disagreements, and conflicting results (12). The field of teleophthalmology now has more options thanks to recent developments in remote and home monitoring OCT technology.

The most popular NVHO system utilizes a specially designed SD OCT device intended for home use, enabling patients to conduct self-imaging with OCT technology regularly. This compact device is tailored for commercial application. Users can adjust the height of the device and choose which eye to scan. An innovative proprietary automatic feedback system assists in guiding head positioning and visual fixation by providing prompts for proper alignment and instructing users to focus on a blinking target during the scanning process.

Upon a user’s initial engagement with the device, it undergoes a one-time automatic calibration procedure that personalizes imaging in accordance with the individual’s refractive error and axial length. The NVHO scan consists of a horizontal raster comprising 88 B-scans across an area measuring 3 × 3 mm (10 × 10 degrees field of view), precisely centered on the point of fixation of the eye. Key specifications include a central wavelength of 830 nm, a scanning speed of 10,000 A-scans per second, and each B-scan containing 500 A-scans. After completing each self-imaging session, data is automatically transmitted to the Notal Health Cloud through an integrated cellular modem. The raw data is then utilized to reconstruct cube scans, which are accessible for remote evaluation by physicians or other qualified healthcare professionals through a web-based viewer (10).

Another reported device—spOCT was specifically designed for handling by patients and not by health care professionals in order to allow automatic image acquisition in patients’ homes or in nursing homes. It was tested in the office conditions. The novel volume scan protocol was established to investigate extremely fast volume scanning capabilities, generating an optical specimen of a cube 3.8 × 3.8 mm with resolutions of 50 × 50 pixels, 100 × 100 pixels, or 150 × 150 pixels; a scan depth of 4.2 mm; and a depth resolution of 2,048 pixels (13).

The third model that has already been tested is SELFF-OCT creating an OCT volume scan of the central retina without the need for a beam scanner and other expensive components such as spectrometers or tunable light sources by using full-field technology. It uses an extended illumination of the retina by a 0.9 mW parallel beam from a superluminescent diode with a 840 nm wavelength and a 26 nm spectral bandwidth. It records a densely sampled volumetric retina scan of a lateral area of 4.5 × 1.4 mm axial resolution of 12 μm and a horizontal resolution of about 17 μm (14).

However, a thorough assessment of these technologies’ diagnostic precision, clinical use, safety and patient outcomes is still required. The primary objective of this study was to evaluate the diagnostic accuracy of home monitoring OCT devices and remote OCT technology compared to standard in-office OCT in teleophthalmology settings across various eye conditions. The secondary objectives were to evaluate the feasibility, safety, and clinical utility of using these remote and home-based OCT technologies for remote disease monitoring and management. It will fill up the gaps that now exist, offer a thorough grasp of the advantages and restrictions of using home/remote OCT in teleophthalmic practices, and pinpoint the elements that contribute to the effective application of home monitoring/remote OCT in teleophthalmology (15). This review seeks to improve clinical decision-making, direct future research paths, and further the continuous advancement of tele ophthalmic practices by bringing together the available information. Additionally, it can help shape best practices and recommendations for the efficient application of home monitoring/remote OCT in teleophthalmology, guaranteeing that patients, wherever they may be in the world, will receive top-notch care.

### Research question

What is the diagnostic accuracy, feasibility, and clinical utility of home monitoring OCT devices and remote OCT technology compared to standard in-office OCT in teleophthalmology settings for the management of various eye diseases?

## Materials and methods

The “Cochrane Community” criteria and recommendations for Preferred Reporting Items for Systematic Reviews and Meta-Analysis were followed in the conduct of this investigation (16).

### Literature search and study selection

A manual and electronic search of publications from various databases, such as, PubMed, ScienceDirect, Google Scholar and the Cochrane Library, published from January 2004 up to May 2024, was done. The search was restricted to human-only articles and studies published in English. The following search strategy was first used in PubMed before modified to be used in in the other databases: ((“Optical Coherence Tomography”[MeSH] OR “optical coherence tomography”[tiab(Title/Abstract tag)]) AND (“Telemedicine”[MeSH] OR “teleophthalmology”[tiab] OR “remote eye care”[tiab] OR “home OCT”[tiab] OR “self-imaging”[tiab])). Also thoroughly checked were the reference lists of the identified articles to find any additional relevant articles. All studies retrieved from the databases were exported to EndNote X9 software for screening. First duplicate studies were removed and before screening of the title and abstracts of the studies. Thereafter full-text review followed and studies that met the inclusion criteria were included in the review. The screening at all stages done independently by two authors and discrepancy were resolved through a third party.

### Inclusion criteria

Only articles that had been published between January 1, 2004, and May 28, 2024, and written in English were included. The following requirements had to be met for the articles to be considered in this review:

1. Randomized controlled trials (RCTs). Also, cohort studies, case–control studies, and observational studies were included.
2. Studies utilizing home monitoring/remote optical coherence tomography (OCT) in teleophthalmology settings.
3. For studies to be considered, they included patients (aged 18 years and above) diagnosed with eye disorders conditions.
4. Studies reporting outcomes related to diagnostic accuracy (sensitivity, specificity), safety, and clinical utility.

### Exclusion criteria

1. Reviews, editorials, study protocols, conference papers, commentaries on published articles, case studies, and studies with insufficient data.
2. Studies that did not have the relevant outcome measures.
3. Studies not involving home monitoring/ remote OCT in teleophthalmology settings or using other imaging techniques.
4. Outdated studies (published before 2004) and studies published in other languages.

### Quality assessment

The papers that satisfied the eligibility criterion were assessed for methodological quality. The quality of the included studies was evaluated using the “CASP Checklist” recommended by the Cochrane Collaboration qualitative methodologies groups (17). The 10 questions that make up the CASP tool assessment analyze the rigor of the research process in addition to the validity and applicability of the key findings (18). For this system, no specific ranking system was developed. However, according to Butler et al. (18), the qualitative scores for each evaluated item might be “Yes” (1 point), “Cannot tell” (0.5 points), or “No” (0 points). Consequently, a paper was categorized as “High” quality if “Yes” was indicated in at least two-thirds of the CASP sections; as “Moderate” quality if the score was between four and six “yeses”; and as “Low” quality if more than two-thirds of the responses were “No.” Two authors independently completed this and any discrepancies were resolved through a third party.

### Data extraction

Data extraction came next after selection of the studies and appraisal of their quality. The following data was congregated using a standardized Microsoft Excel data collection form l: Author and year of publication, study design, research settings, sample size, participants feature (age and gender), type of OCT device, and study’s measurable outcome (s). These stages were independently completed by two authors and disagreement between the two authors were resolved through a third party. Two authors completed this task independently and any discrepancies were resolved through a third party.

### Data synthesis

Due to the wide variations in methodology, different type of home monitoring/remote OCT devices, differences in measurements for the outcomes and insufficient data, a quantitative synthesis of the data was not feasible. The results from the studies were therefore analyzed using thematic and narrative analysis, as defined by Braun and Clarke (19). Thematic analysis has the advantage of enabling researchers to make interpretations based on recurrent themes in seemingly distinct studies and offer results that unswervingly support health practitioners (19).

## Results

### Search results

**Figure 1:**
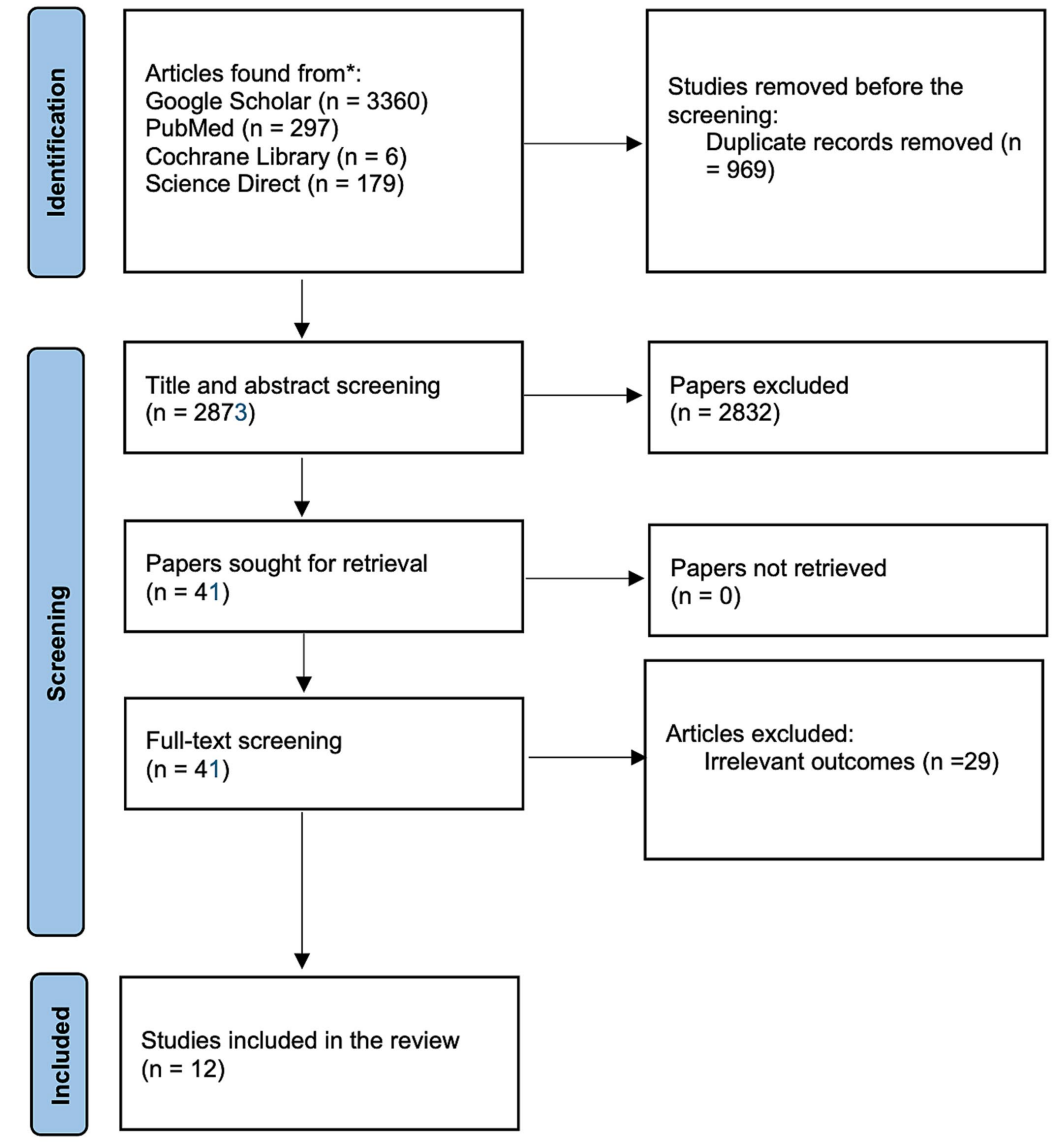
The process for selecting study according to PRISMA.

The literature search from the electronic data base resulted into a total of 3,842 items. There were 3,360 publications found by Google Scholar, 6 trials found by Cochrane, 297 articles found by PubMed, and 179 papers found by ScienceDirect. Nine hundred sixty-nine duplicates were discarded before the screening of titles and abstracts. Title and abstract screening resulted in the exclusion of 2,832 articles. They either adhered to the research design specified in the exclusion criteria or failed to report on the home monitoring/ remote OCT in teleophthalmology settings. Only 12 of the remaining 41 articles fully complied with the inclusion criteria after being read in their entirety. The process selection of studies is presented in a flowchart in Figure 1.

### Study characteristics

The main features of included studies are presented in Table 1. These studies were published between 2011 and 2022. The sample size of these studies ranged from 4 to 1,257 with a total of 3,539 participants. All these participants were adults of ages ranging from 35 years and above. The other characteristics of the included studies are summarized in Table 1.

**Table 1:** Characteristics of the included studies.

Study ID	Study design	Study sample	Study settings	Sample features (age, gender)	Diseases detected	Intervention (type of OCT used)	Outcome measures
Anton et al. (22)	Population-based study	1,006	Spain/remote	Women and men; mean age of 67 ± 7.8 years	Glaucoma	Portable SD-OCT (iVue)	Image quality, screening results
Liu et al. (11)	Cohort study	1,257	China/remote	Men and women; mean age of 64.2 years	Retinal diseases	OCT-AI (iScan)	Sensitivity and specificity
Maa et al. (24)	Cohort study	256	United States/remote	Men and women; mean age of 60 years	Glaucoma or retinal diseases	TECS protocol with OCT (in-office)	Diagnostic accuracy, sensitivity, specificity
Kelly et al. (20)	Cohort study	50	United Kingdom/remote	Men and women aged 35 years and above	Macular diseases	Topcon 3D OCT-200	Specificity
Keenan et al. (23)	Observational longitudinal study	4	Israel/home	Men and women; mean age of 73.8 years (69–80 years)	Age-related macular degeneration	Notal Vision Home OCT (NVHO)	Satisfactory quality, self-imaging completion
Maloca et al. (13)	Prospective study	31	Switzerland/office	Men and women; mean age of 79.6 years (69–92 years)	Age-related macular degeneration	sparse OCT (spOCT, MIMO_02)	Feasibility and safety
von der Burchard et al. (14)	Prospective single-arm diagnostic accuracy study	46	Ireland/office	Men and women; mean age of 79 years (57–92 years)	Age-related macular degeneration	Self-examination low-cost full field OCT (SELFF-OCT)	Successful self-scan rate, sensitivity, specificity
Liu et al. (10)	Prospective observational study	15	United States/home	Men and women; mean age of 73.4 (57 to 81 years)	Age-related macular degeneration	Notal Vision Home OCT (NVHO)	Weekly self-scan rate, image quality, scan duration
Liu et al. (25)	Cross-sectional study	475	China/office	Men and women; mean age of 58.3 years	Retinal diseases	Self-imaging OCT (a prototype device)	Feasibility and diagnostic accuracy
Nahen et al. (26)	Cohort study	69	United States/office	Men and women with mean age of 79.5 years	Age-related macular degeneration	Notal Vision Home OCT (NVHO)	Diagnostic accuracy, safety and feasibility
Kim et al. (21)	Cross-sectional study	290	Israel/office	Men and women; mean age of 78.8 years	Retinal diseases	Notal Vision Home OCT (NVHO)	Feasibility, safety, diagnostic accuracy
Blinder et al. (27)	Prospective observational study	40	United States/home	Men and women; aged between 69–83 years	Age-related macular degeneration	Notal Vision Home OCT (NVHO)	Feasibility, diagnostic accuracy, scan frequency and duration

### Quality assessment

The articles included in the theme synthesis generally had Moderate methodological quality (mean quality = 6.25). Eight studies (66.7%) depicted a moderate quality in the overall score, while 4 papers (33.3%) had a high-quality overall score. Different Scores were exhibited for Items 3 and 6. Item 3 concerns whether the search design was suitable in relative to its objectives, and 6 studies did not to meet this requirement. Item 6, which is concerned with the influences in the affiliation between study participants and researchers, was only excellently addressed by only two studies (20, 21). Table 2 provides more details on the quality appraisal of the included studies (see Table 3).

**Table 2:** Quality Assessment results for the included studies.

Study	Item 1	Item 2	Item 3	Item 4	Item 5	Item 6	Item 7	Item 8	Item 9	Item 10	Score	Classification of quality
Anton et al. (22)	Y	Y	Y	Y	Y	N	Y	Y	Y	Y	8	High
Liu et al. (11)	Y	Y	N	Y	N	N	Y	Y	Y	Y	7	High
Maa et al. (24)	Y	Y	N	N	Y	N	Y	Y	N	Y	6	Moderate
Kelly et al. (20)	Y	N	Y	Y	Y	Y	Y	N	Y	Y	8	High
Keenan et al. (23)	Y	Y	N	Y	N	N	Y	N	Y	Y	6	Moderate
Maloca et al. (13)	Y	N	Y	Y	N	N	Y	N	Y	Y	6	Moderate
von der Burchard et al. (14)	Y	N	N	N	Y	N	Y	N	Y	N	4	Moderate
Liu et al. (10)	N	Y	N	Y	N	N	Y	Y	N	Y	5	Moderate
Liu et al. (25)	Y	N	N	N	Y	N	Y	Y	N	Y	5	Moderate
Nahen et al. (26)	Y	N	Y	Y	N	N	Y	N	Y	Y	6	Moderate
Kim et al. (21)	Y	N	Y	Y	Y	Y	Y	N	Y	Y	8	High
Blinder et al. (27)	Y	Y	N	Y	N	N	Y	N	Y	Y	6	Moderate

Y = Yes; N = No, Item 1 = Clear statement of aim, Item 2 = Appropriate qualitative methodology, Item 3 = Appropriate research design, Item 4 = Sampling, Item 5 = Data collection, Item 6 = Researcher reflexivity, Item 7 = Ethical consideration, Item 8 = Appropriate data analysis, Item 9 = Clear statement of findings, Item10 = Research value.

**Table 3:** Diagnostic accuracy and data collection techniques from the selected studies.

Paper	Diagnostic accuracy	Data collection
Anton et al. (22)	Interobserver agreement outcomes were moderate to good with a kappa coefficient of 0.37 and PABAK index of 0.58 Kappa and PABAK values between OCT and photographs were 0.52 and 0.82 for the first evaluation Fair-good quality images and useful images was obtained with OCT [962 (97.2%) and 946 (94%), respectively] compared with fundus photographs [945 (95.5%) and 927 (92.1%), respectively]	The remote center where examination and data collection took place was located at a primary care center Finally, the web-based telemedicine platform (DYSEO) automatically generated a report based on the signs identified and ratings performed by the evaluators
Liu et al. (11)	Sensitivity of 96.6% (95% CI, 91.8–98.7%) and specificity of 98.8% (95% CI, 98.0–99.3%) for detecting urgent cases Sensitivity of 98.5% (95% CI, 96.5–99.4%) and specificity of 96.2% (95% CI, 94.6–97.3%) for detecting both urgent and routine cases	OCT-AI-based telemedicine platform deployed at primary care stations. 2 ophthalmologists jointly graded the data set collected from this pilot application
Maa et al. (24)	Reader 1: Sensitivity (95% CI) for glaucoma: 0.74 (0.61, 0.84), macular degeneration: 0.50 (0.12, 0.88) and diabetic retinopathy: 0.75 (0.35, 0.97) Specificity (95% CI) for glaucoma: 0.84 (0.77, 0.89), macular degeneration: 0.99 (0.97, 1.00) and diabetic retinopathy:0.99 (0.97, 1.00) Reader 2: Sensitivity (95% CI) for glaucoma: 0.37 (0.25, 0.49), for macular degeneration: 0.67 (0.22, 0.96) and diabetic retinopathy: 0.63 (0.24, 0.91) Specificity (95% CI) for glaucoma: 0.82 (0.76, 0.88), for macular degeneration: 0.95 (0.91, 0.97) and for diabetic retinopathy: 0.99 (0.97, 1.00)	In Technology-Based Eye Care Services (TECS) protocol patients’ data were loaded into a secure research database, REDCap. OCT images were interpreted by 2 readers. Readers were blinded to the patient’s data and to each other’s interpretations After a 3 month wash out period, images were randomly selected for a second read. Readers, blinded to their initial read, repeated the same procedure, and re-documented their findings on REDCap case report forms
Kelly et al. (20)	In all cases the community SD OCT image quality was considered by the ophthalmologist to be as good as, or better than, the SD OCT images captured by the hospital nursing staff. In two cases the community SD OCT image was superior to that captured in hospital clinic on initial attempt	Teleophthalmology consultation based on SD-OCT images acquired by the community optometrist and transmitted to hospital eye services
Keenan et al. (23)	Of the 211 scans, the proportion for which the NOA grading agreed with the human grading was 94.7%. The equivalent values for IRF and SRF were 97.6 and 93.3%, respectively Of the 93 scans with retinal fluid present, 93.5% were graded by the NOA as having fluid present Of the 118 scans with no retinal fluid, 95.7% were graded by the NOA as having no fluid	The participants were asked to perform self-imaging using the device on each study eye daily for 1 month. The imaging data were uploaded automatically to the Notal Health Cloud The duration of the self-imaging acquisition and the MSI were recorded The home OCT scans underwent evaluation separately by the NOA and human expert graders
Maloca et al. (13)	The difference in the CRT measurements obtained using the two devices was not statistically significant (paired *t*-test, two-sided, *t* 1/4 1.7198, df 1/4 57, *p* 1/4 0.091) Intrareader reliability coefficient of 0.968 (lower bound of one-sided 95% CI: 0.935) and an interrater reliability coefficient of 0.958 (lower bound of one-sided 95% CI: 0.917) The individual intrareader reliabilities for each rater with their lower bound of a one-sided 95% CI were 0.973 (0.940), 0.947 (0.885), and 0.987 (0.971)	spOCT performed in office and the retinal thickness on the spOCT scans was measured manually by one grader using custom software written in MATLAB The quantitative OCT analysis of the reference device was performed manually by a second, independent grader using the manufacturer’s built-in caliber software tool (Spectralis HRA)
von der Burchard et al. (14)	Sensitivity of 0.94 (95% CI 0.79 to 0.99) and a specificity of 0.95 (95% CI 0.82 to 0.99)	SELFF-OCT measurements in clinical settings The patient performed 2 entire measurement cycles (M1 and M2) without medical assistance After finishing the SELFF-OCT measurements, all patients received a detailed scan by a reference SD-OCT (Heidelberg Spectralis HRA + OCT2)
Liu et al. (10)	88% imaging success rate 97% achieved satisfactory quality	The participants performed self-imaging at home on a daily basis for a period of 3 months
Liu et al. (25)	Self-imaging OCT images had consistent CST with SD-OCT, with a mean difference of 0.1 ± 7.7 μm for normal eyes, 4.9 ± 10.6 μm for macular oedema, −1.3 ± 9.5 μm for choroidal neovascularisation, 5.0 ± 7.8 μm for epiretinal membrane Interdevice *κ* values ranged for detecting various retinal lesions ranged from 0.8 to 1.0 Mean (SD) difference of 2.0 (9.4) μm (95% LOA, −16.4 to 20.4 μm) in central subfield thickness (CST) measurements	All participants underwent OCT imaging with both the self-imaging OCT and the SD-OCT (Spectralis, Heidelberg Engineering) on the same day The self-imaging was conducted in a separate examination room within the clinic to simulate a home environment
Nahen et al. (26)	PPA and NPA of the detection of intraretinal and subretinal fluid by the two NVHO systems in comparison to the commercial OCT system was determined The 95% confidence interval of PPA and NPA was derived using the binomial distribution PPA and NPA for detection of fluid, intraretinal fluid, and subretinal fluid in NHVO images was 97/95%, 96/94% and 100/98%, respectively, when compared to commercial OCT systems. The average total CV% for the entire data set was 12.4%, and the mean MSI for the four self-performed images was 3.5, 3.5, 3.6, and 3.5, respectively	In-office screening visits Subjects completed one self-performed practice scan and then a session with up to four self-performed scans in order to provide data on repeatability
Kim et al. (21)	PPA and NPA of the two NVHO systems’ detection of intraretinal and subretinal fluid The 95% CI of the PPA and NPA were derived using the binomial distribution For the NVHO 2.5 device, PPA and NPA for detecting the presence of any fluid (SRF and/or IRF), SRF, and IRF were 98%/96, 93%/96, and 91%/98%, respectively This diagnostic accuracy had agreement rates (PPA and NPA and ORA) of 0.83–1.0 across all VA levels For the NVHO 3 device PPA/ NPA were 97%/95, 96%/94 and 100%/98%, respectively Diagnostic accuracy had accuracy rates (average of PPA and NPA) of 0.71 to 0.94 across all VA levels The overall mean CV% for that entire dataset was 12.4%, and the mean MSI for the four self-images were 3.5, 3.5, 3.6, and 3.5, respectively	Patients used either the NVHO 2.5 or NVHO 3 models in an ophthalmic clinic Data was transferred to a laptop for image processing, mimicking transmission to the cloud as planned when installed at patient’s homes
Blinder et al. (27)	The average MSI was 4.5 (range, 0.6–7); 99.3% had an MSI 2 (“good quality”) NOA successfully quantified fluid on 86.5% of scans	Participants were performing self-imaging et home on both eyes daily for approximately 6 months Home OCT image files were transmitted automatically to the NVMC for processing In-office OCTs were uploaded to a central Reading Center

PABAK, prevalence-adjusted bias-adjusted kappa; CI, confidence interval; MSI, manufacturer signal quality index; PPA, positive percent agreement; NPA, negative percent agreement; CV, coefficient of variation; LOA, limits of agreement; SELFF-OCT, self-examination low-cost full-field OCT; OCT, optical coherence tomography; SD-OCT, spectral domain-OCT; spOCT, sparse OCT; NVHO, Notal Vision Home OCT; NOA, Notal OCT Analyzer.

### Analysis and consistent themes

The recurrent themes in all the included papers were found, evaluated, and analyzed using thematic analysis (19). The results of the included research articles were assessed in conjunction with the reports from the included studies. This was carried out to assess the feasibility, utility, safety, and diagnostic accuracy of employing remote OCT and home monitoring OCT in teleophthalmology. The most prevalent themes are presented as follows.

### Image quality

Data on the image quality of remote OCT and home-monitoring OCT in teleophthalmology were obtained from two studies (22, 23). Compared to fundus photography (95.5% fair-good quality, 92.1% useful images), the remote OCT device produced a higher percentage of fair-good quality images (97.2%) and usable images in screening glaucoma disease (94%) (22). Also, according to Anton et al. (22), there was a substantial rise in the frequency of low-quality photos as participant age increased (*p* < 0.0001). 97.6% of AMD patients’ at-home self-imaging attempts utilizing a home OCT device had acceptable quality for evaluation (23).

### Diagnostic accuracy (sensitivity, specificity)

The research utilizing OCT-artificial intelligence (AI)-driven telemedicine systems implemented in primary care facilities demonstrated a sensitivity of 96.6% and a specificity of 98.8% for urgent case identification. Furthermore, it achieved a sensitivity of 98.5% and a specificity of 96.2% when identifying both routine and urgent retinal disease cases. This pilot program, integrated with AI, significantly alleviated the burden on human consultations by 96.2% for a substantial volume of normal cases. The platform facilitated online medical recommendations for detected disease cases within an average timeframe of 21.4 h (11).

Collaboration between primary and secondary healthcare sectors regarding the implementation of retinal imaging technology, with e-referrals using OCT, was evaluated in the United Kingdom. In this context, community optometrists often serve as the primary source for referrals. Based on remote OCT scans, telemedicine consultations were successful in 96% of instances, yielding a workable diagnosis and treatment plan for retinal disease the following business day (20).

In a separate investigation, the sensitivity and specificity of the most prevalent eye conditions were assessed by comparing the Technology-Based Eye Care Services (TECS) Protocol conducted by a technician using OCT with traditional Face-To-Face (FTF) examinations. The study was conducted involving the veteran population in the United States, with a total of 256 patients recruited for the research. Specificity rates for cataracts, glaucoma, macular degeneration, and diabetic retinopathy ranged from 0.84 to 0.99 for 2 evaluators. Over the course of the diagnostic categories, sensitivity estimates showed greater variability, ranging from 0.50 to 1.00 for one reader and 0.37 to 0.90 for the other reader (24). Diagnostic precision in the decision-making process for patients with neovascular age-related macular degeneration (AMD) was evaluated in a study conducted in Ireland by von der Burchard et al. (14). This research utilized a specially developed self-examination low-cost full-field optical coherence tomography (SELFF-OCT). The authors demonstrated that SELFF-OCT exhibited a sensitivity of 0.94 and specificity of 0.95 regarding therapy decisions for AMD, when compared to standard OCT.

Another cross-sectional study carried out in China assessed the accuracy and consistency of measuring central subfield thickness (CST) using self-imaging OCT among patients with retinal disorders. Liu et al. (25) reported that all 160 participants (100%) successfully completed the self-imaging process, yielding interpretable images from the self-imaging OCT devices. The authors noted a strong correlation between the NVHO scans and those obtained in-office, achieving a Pearson correlation coefficient of *r* = 0.90 for measurements of CST. Other studies indicate that a significant proportion of high-quality scans can be achieved with home-use OCT. Most of these investigations focus on the NVHO application and have taken place in Israel and the United States. Keenan et al. (23) found that from 240 attempts at self-imaging, an impressive 87.9% were completed successfully, with 97.6% of the resulting self-scans meeting quality standards. Similarly, Nahen et al. (26) reported that all participants, encompassing 69 eyes (93%), successfully conducted self-imaging using the NVHO. Kim et al. (21) observed comparable outcomes; their research demonstrated successful imaging in 88% of the eyes among 264 out of 290 study participants utilizing the NVHO. Additionally, Blinder et al. (27) identified that out of 2,304 scans performed, 86.5% were suitable for fluid quantification.

### Direct comparison of remote/home OCT with conventional OCT

As per Liu et al. (10), there was a 96% agreement on the presence or absence of fluid in the 6 × 6 mm OCT scans rated by retinal specialists in the office and the 3 × 3mm OCT scans graded at home using automated analysis software. Furthermore, Liu et al. (10) discovered a strong correlation (*r* = 0.90) between CST measurements from in-office OCT scans and software-analyzed home OCT scans.

The findings of von der Burchard’s et al. (14) investigation in 2022 showed that, although SELFF-OCT had a smaller field of view and a lower signal-to-noise ratio, it nonetheless revealed the same structures in individuals with AMD when compared to conventional office OCT.

Comparing telemedicine consultations based on remote OCT pictures to conventional in-person procedures, Kelly et al. (20) discovered that the latter took longer to review referrals and deliver a working diagnosis.

The convectional OCT device and the small sample yielding sparse OCT (spOCT) produced similar cross-sectional OCT picture information; however, because the spOCT was intended for rapid acquisition rather than maximum resolution, the images seemed more pixelated and grainier (13). Additionally, compared to spOCT, the choroid and vitreous were easier to identify in convectional OCT. (13) This was one of the main differences observed between spOCT and the standard OCT.

Maa et al. (24) reported that differences were seen in the diagnostic categories between the TECS-OCT protocol and FTF visits. The kappa statistics indicated a moderate to substantial agreement in these findings.

Strong correlation coefficients (0.948 to 0.999) were found for the selected cohort, with mean (SD) differences in CST between self-imaging OCT and standard OCT ranging from 0.1 μm to 5.0 μm for various eye conditions (25). Furthermore, in a subsequent cohort, there was a significant and consistent correlation in the measures of CST (correlation coefficient = 0.998), with a mean (SD) difference in CST between the two devices of 2.0 (9.4) μm (25).

Compared to the in-office commercial OCT, Nahen et al. (26) found that the positive percent agreement and negative percent agreement for detection of fluid, intraretinal fluid, and subretinal fluid in at least one of three consecutive NVHO images was 97/95%, 96/94% and100/98%, respectively. In the study by Kim et al. (21), 99% of the images from the NVHO were deemed gradable by the reading ophthalmologist compared to 99.8% by commercial OCT.

In comparing NVHO and with an in-office OCT, Blinder et al. (27) found that for 35 scan pairs detected as having fluid by in-office OCT, the NVHO detected fluid on 31 scans (89%) and for 14 scan pairs detected as having no fluid on in-office OCT, the NVHO did not detect fluid on 10 scans (71%).

### Feasibility and safety

According to Liu et al. (10), 97% of participants claimed that self-OCT scanning was simple and convenient. Overall, participants gave NVHO positive feedback in this study. Similarly, two other reports revealed that more than 95% of participants either agreed or strongly agreed that the NVHO was simple and comfortable (21, 26). Furthermore, Blinder et al. (27) reported that all subjects agreed that scanning using NVHO became easier overtime.

In a different study, Liu et al. (25) discovered that participants were largely happy with the self-imaging OCT, agreeing with 89% of the claims on its comfort and simplicity and 73% of them saying they would want to use it for further monitoring.

Maloca et al. (13) indicate that there were no safety issues with the small sample yielding sparse OCT acquisition (spOCT) and that patient discomfort was negligible.

Blinder et al. (27) reported that all subjects agreed that scanning using NVHO became easier overtime while two other studies reported that more than 95% of participants either agreed or strongly agreed that the NVHO was simple and comfortable (21, 26).

### Scan duration/rate for home OCT

The self-OCT study lasted 91.3 ± 9.5 days on average, and throughout that period, each study eye attempted 86 scans on average (10).

According to Liu et al. (25), the self-imaging OCT had a mean (SD) scanning time of 66.7 ± 20.1 s per eye, which was much faster than the standard OCT’s 73.3 ± 32.5 s per eye.

Blinder et al. (27) reported that NVHO had a mean (standard deviation) of 6.3 (0.6) for weekly scanning frequency and 47 (17) seconds for scan duration per eye.

## Discussion

This systematic review summarized the available data on the clinical usage, feasibility and diagnostic accuracy (sensitivity and specificity) of remote OCT technology and home monitoring OCT devices in teleophthalmology for a range of ocular disorders such as AMD, glaucoma, diabetic retinopathy etc. There were 12 studies with 3,539 participants included in this review. The main conclusions were that home monitoring and remote OCT devices show good image quality, high diagnostic accuracy (sensitivity and specificity) when compared to OCT performed in an office, and adequate patient acceptability and safety.

The utilization of OCT devices for home and remote monitoring through telemedicine is still in its formative phase. Currently, there is a limited pool of data, accompanied by a relatively modest body of evidence; however, the advantages associated with these teleophthalmic approaches are becoming increasingly apparent. The existing research, albeit sparse, plays a crucial role in enhancing our understanding of the emerging fields of home monitoring utilizing OCT technology and teleophthalmology. Despite the limited resources available, this review underscores the significant promise within the swiftly advancing realm of telemedicine concerning OCT studies, particularly emphasizing its notable precision in disease screening, diagnosis, and ongoing monitoring.

Overall, the studies discovered that most of the time, both at-home and remote OCT devices generate pictures clear enough for monitoring and diagnosis. For instance, using a portable OCT device, Anton et al. reported 97.2% fair-good quality images; using a home OCT system (22), Keenan et al. (23) discovered 97.6% satisfactory image quality. The excellent quality makes it possible to evaluate retinal architecture and disease biomarkers with accuracy. This fits with recent studies that demonstrate home and remote OCT can produce pictures that are as good as those obtained from in-office OCT in terms of pathology detection (13, 14). While patient self-operation may increase the likelihood of image artifacts, advancements in OCT technology and automated analysis assist guarantee diagnostic-grade images.

Several studies showed that for diagnosis and treatment decisions, home/remote OCT and traditional in-office OCT agreed quite well. Excellent correlation was found between in-office and at-home OCT in central subfield thickness measures (10, 25). In addition, there was a 96% agreement between the results of home OCT scans evaluated by AI software and clinician reading of in-office scans for fluid detection (10, 21, 26). Several studies indicated that home/remote OCT screening has excellent sensitivity and specificity when compared to in-person evaluation (11, 24). However, there was variability between conditions and interpreters. Overall, these results confirm that conducting OCT assessments remotely or at home can offer accurate diagnostic and monitoring prospects equivalent to those of traditional in-person appointments.

Evidence from the studies also showed that patient-operated home OCT devices demonstrated effective utility and safety alongside precise diagnostic results. Self-OCT scanning was easy, convenient, and comfortable for the majority of patients (10, 11, 21, 26, 27). The home-based OCT procedure was conducted efficiently and without any adverse reactions. The preference for home OCT over office-based OCT is enhanced due to quicker scan times facilitated by home equipment. The practicality of home OCT devices for remote monitoring is supported by patients’ favorable experiences and their ability to operate them correctly.

The findings of this study are consistent with earlier research showing the viability of OCT-based teleophthalmology. According to Rathi et al. (4), mobile OCT demonstrated a high diagnosis accuracy for main retinal disorders when used during screening camps. The current analysis adds to the mounting body of data that OCT technology, whether at home or remotely, can now produce imaging sufficient for reliable telediagnosis. The results also support earlier studies that shown the viability of OCT outside of the office.

All this is important in view of the aging population, which is associated with an increased incidence of glaucoma and AMD. The occurrence of AMD has increased at a rate 2.75 times higher than the projections established in 2011 (28). The implications of AMD extend beyond health, imposing significant financial burdens that encompass time, monetary expenses, and overall quality of life. Annually, the financial impact of AMD ranges between approximately $8,814 and $23,400, escalating to between $32,491 and $70,200 after 3 years of treatment (29).

The prompt commencement of treatment, alongside ongoing oversight by an ophthalmologist, is essential for safeguarding vision and minimizing the risk of significant visual impairment. The onset of blindness frequently leads to substantial social care expenses and a diminished quality of life.

In a standard ophthalmology practice, a range of diagnostic assessments is available to facilitate the identification of disease progression. These include OCT, comprehensive fundus examinations, Amsler grid testing, best-corrected visual acuity evaluations, and self-reported perceived changes in vision. Quality-adjusted life years (QALYs) serve as a crucial metric for assessing the cost-effectiveness of these diagnostic procedures (30). Hernandez et al. (31) estimated the expected QALYs and costs of 5 different index tests to evaluate which modality of detecting early conversion is the most cost effective. Results showed SD-OCT generated a higher QALY (5.830) followed by fundus assessment (5.787), Amsler grid (5.736), patients’ subjective vision (5.630), and visual acuity (5.600). Healthcare-associated costs were also lower compared to other forms of detection, with SD-OCT costing a yearly average of £19,406 per patient ($24,681 in 2024 USD). This is likely due to the high sensitivity of SD-OCT to detect nAMD, allowing for earlier initiation of treatment (31).

The findings of this analysis confirm that it is feasible to move some aspects of ophthalmic care to telemedicine platforms that use remote, or at-home OCT. Patients will benefit from better access and convenience as a result of the ability to precisely diagnose and monitor illnesses remotely. The results suggest that virtual visits and at-home OCT monitoring should be part of the treatment plans for long-term eye conditions such as diabetic retinopathy and glaucoma. More broadly, the results support the creation of comprehensive programs in teleophthalmology that completely integrate remote OCT technologies. Supporting coverage and reimbursement mechanisms can help encourage the use of teleophthalmology services by policymakers. It could be necessary to update guidelines to reflect approved applications of telemedicine and remote OCT.

## Limitations of the study

The current study has several limitations, such as limited study sample sizes, a dearth of randomized controlled trials, differences in the study populations, and the OCT technology evaluated. Also, while no major safety issues were reported, there may be minimal risks associated with home OCT use that were not captured in the included studies. It is necessary to do more sizable prospective studies that directly compare in-office OCT with home/remote OCT using standardized measures. It is also necessary to conduct long-term investigations that use remote OCT monitoring to track clinical results. Future studies ought to assess how cost-effective it is to use remote or at-home OCT.

## Conclusion

In conclusion, current data shows that both home/remote OCT devices can effectively deliver high-quality diagnostic imaging to assist precise diagnosis, remote clinical management, and disease monitoring. To further characterize the effects on clinical and patient-reported outcomes over time, more extensive and rigorous comparative effectiveness studies are still required. In order to improve patient care and eye health outcomes, the results generally justify the use of remote OCT systems in full teleophthalmology programs.

## References

[1] Flaxman SR Bourne RRA Resnikoff S Ackland P Braithwaite T Cicinelli MV . Global causes of blindness and distance vision impairment 1990–2020: a systematic review and meta-analysis. Lancet Glob Health. (2017) 5:e1221–34. doi: 10.1016/S2214-109X(17)30393-5, PMID: 29032195

[2] World Health Organisation. World report on vision. (2019). Available at: https://www.who.int/docs/default-source/documents/publications/world-vision-report-accessible.pdf?sfvrsn=223f9bf7_2 (Accessed February 7, 2024).

[3] Jin K Lu H Su Z Cheng C Ye J Qian D. Telemedicine screening of retinal diseases with a handheld portable non-mydriatic fundus camera. BMC Ophthalmol. (2017) 17:89. doi: 10.1186/s12886-017-0484-5, PMID: 28610611 PMC5470179

[4] Rathi S Tsui E Mehta N Zahid S Schuman JS. The current state of Teleophthalmology in the United States. Ophthalmology. (2017) 124:1729–34. doi: 10.1016/j.ophtha.2017.05.026, PMID: 28647202 PMC6020848

[5] Sharafeldin N Kawaguchi A Sundaram A Campbell S Rudnisky C Weis E . Review of economic evaluations of teleophthalmology as a screening strategy for chronic eye disease in adults. Br J Ophthalmol. (2018) 102:1485–91. doi: 10.1136/bjophthalmol-2017-311452, PMID: 29680803

[6] Dolar-Szczasny J Barańska A Rejdak R. Evaluating the efficacy of teleophthalmology in delivering ophthalmic care to underserved populations: a literature review. J Clin Med. (2023) 12:3161. doi: 10.3390/jcm12093161, PMID: 37176602 PMC10179149

[7] Chopra R Wagner SK Keane PA. Optical coherence tomography in the 2020s—outside the eye clinic. Eye. (2021) 35:236–43. doi: 10.1038/s41433-020-01263-6, PMID: 33168975 PMC7853067

[8] Mallipatna A Vinekar A Jayadev C Dabir S Sivakumar M Krishnan N . The use of handheld spectral domain optical coherence tomography in pediatric ophthalmology practice: our experience of 975 infants and children. Indian J Ophthalmol. (2015) 63:586–93. doi: 10.4103/0301-4738.167108, PMID: 26458476 PMC4652249

[9] Sahu A Oh Y Peterson G Cordova M Navarrete-Dechent C Gill M . *In vivo* optical imaging-guided targeted sampling for precise diagnosis and molecular pathology. Sci Rep. (2021) 11:23124. doi: 10.1038/s41598-021-01447-4, PMID: 34848749 PMC8633337

[10] Liu Y Holekamp NM Heier JS. Prospective, longitudinal study: daily self-imaging with home OCT for neovascular age-related macular degeneration. Ophthalmol Retina. (2022) 6:575–85. doi: 10.1016/j.oret.2022.02.011, PMID: 35240337

[11] Liu X Zhao C Wang L Wang G Lv B Lv C . Evaluation of an OCT-AI–based telemedicine platform for retinal disease screening and referral in a primary care setting. Transl Vis Sci Technol. (2022) 11:4. doi: 10.1167/tvst.11.3.4 PMC8914565 35254422

[12] Nittari G Savva D Tomassoni D Tayebati SK Amenta F. Telemedicine in the COVID-19 era: a narrative review based on current evidence. Int J Environ Res Public Health. (2022) 19:5101. doi: 10.3390/ijerph19095101, PMID: 35564494 PMC9105428

[13] Maloca P Hasler PW Barthelmes D Arnold P Matthias M Scholl HPN . Safety and feasibility of a novel sparse optical coherence tomography device for patient-delivered retina home monitoring. Transl Vis Sci Technol. (2018) 7:8. doi: 10.1167/tvst.7.4.8, PMID: 30050725 PMC6058910

[14] von der Burchard C Sudkamp H Tode J Ehlken C Purtskhvanidze K Moltmann M . Self-examination low-cost full-field optical coherence tomography (SELFF-OCT) for neovascular age-related macular degeneration: a cross-sectional diagnostic accuracy study. BMJ Open. (2022) 12:e055082. doi: 10.1136/bmjopen-2021-055082, PMID: 35760534 PMC9237881

[15] Ghazala FR Dall’Ozzo S McGowan G IAT L. Teleophthalmology-enabled direct vitreoretinal surgery listing from community optometric practice: enhanced efficiency during coronavirus disease 2019, and beyond? Telemed J E Health. (2021) 27:816–9. doi: 10.1089/tmj.2020.0361, PMID: 33320049

[16] Cumpston MS McKenzie JE Welch VA Brennan SE. Strengthening systematic reviews in public health: guidance in the Cochrane Handbook for Systematic Reviews of Interventions, 2nd edition. J Public Health. (2022) 44:e588–92. doi: 10.1093/pubmed/fdac036, PMID: 35352103 PMC9715291

[17] Noyes J Booth A Flemming K Garside R Harden A Lewin S . Cochrane qualitative and implementation methods group guidance series—paper 3: methods for assessing methodological limitations, data extraction and synthesis, and confidence in synthesized qualitative findings. J Clin Epidemiol. (2018) 97:49–58. doi: 10.1016/j.jclinepi.2017.06.020, PMID: 29247700

[18] Butler A Hall H Copnell B. A guide to writing a qualitative systematic review protocol to enhance evidence-based practice in nursing and health care. Worldviews Evid-Based Nurs. (2016) 13:241–9. doi: 10.1111/wvn.12134, PMID: 26790142

[19] Braun V Clarke V. What can “thematic analysis” offer health and wellbeing researchers? Int J Qual Stud Health Well-being. (2014) 9:26152. doi: 10.3402/qhw.v9.26152 25326092 PMC4201665

[20] Kelly SP Wallwork I Haider D Qureshi K. Teleophthalmology with optical coherence tomography imaging in community optometry. Evaluation of a quality improvement for macular patients. Clin Ophthalmol. (2011) 5:1673–8. doi: 10.2147/OPTH.S26753, PMID: 22174576 PMC3236713

[21] Kim JE Tomkins-Netzer O Elman MJ Lally DR Goldstein M Goldenberg D . Evaluation of a self-imaging SD-OCT system designed for remote home monitoring. BMC Ophthalmol. (2022) 22:261. doi: 10.1186/s12886-022-02458-z, PMID: 35689210 PMC9186475

[22] Anton A Nolivos K Pazos M Fatti G Herranz A Ayala-Fuentes M . Interobserver and intertest agreement in telemedicine glaucoma screening with optic disk photos and optical coherence tomography. J Clin Med. (2021) 10:3337. doi: 10.3390/jcm10153337 34362120 PMC8347319

[23] Keenan TDL Goldstein M Goldenberg D Zur D Shulman S Loewenstein A. Prospective, longitudinal pilot study. Ophthalmol Sci. (2021) 1:100034. doi: 10.1016/j.xops.2021.100034, PMID: 36249303 PMC9562348

[24] Maa AY McCord S Lu X Janjua R Howell AV Hunt KJ . The impact of OCT on diagnostic accuracy of the technology-based eye care services protocol. Ophthalmology. (2020) 127:544–9. doi: 10.1016/j.ophtha.2019.10.025, PMID: 31791664

[25] Liu Z Huang W Wang Z Jin L Congdon N Zheng Y . Evaluation of a self-imaging OCT for remote diagnosis and monitoring of retinal diseases. Br J Ophthalmol. (2023) 108:1154–60. doi: 10.1136/bjo-2023-324012 37903558

[26] Nahen K Benyamini G Loewenstein A. Evaluation of a self-imaging SD-OCT system for remote monitoring of patients with neovascular age related macular degeneration. Klin Monatsbl Augenheilkd. (2020) 237:1410–8. doi: 10.1055/a-1271-6834 33285588

[27] Blinder KJ Calhoun C Maguire MG Glassman AR Mein CE Baskin DE . Home OCT imaging for newly diagnosed neovascular age-related macular degeneration. Ophthalmol Retina. (2024) 8:376–87. doi: 10.1016/j.oret.2023.10.012, PMID: 37879537 PMC10997472

[28] Rein DB Wittenborn JS Burke-Conte Z Gulia R Robalik T Ehrlich JR . Prevalence of age-related macular degeneration in the US in 2019. JAMA Ophthalmol. (2022) 140:1202–8. doi: 10.1001/jamaophthalmol.2022.4401, PMID: 36326752 PMC9634594

[29] Meer EA Oh DH Brodie FL. Time and distance cost of longer acting anti-VEGF therapies for macular degeneration: contributions to drug cost comparisons. Clin Ophthalmol. (2022) 16:4273–9. doi: 10.2147/OPTH.S384995 36578665 PMC9792116

[30] Owens DK. Interpretation of cost-effectiveness analyses. J Gen Intern Med. (1998) 13:716–7. doi: 10.1046/j.1525-1497.1998.00211.x, PMID: 9798822 PMC1497852

[31] Hernandez R Kennedy C Banister K Goulao B Cook J Sivaprasad S . Early detection of neovascular age-related macular degeneration: an economic evaluation based on data from the EDNA study. Br J Ophthalmol. (2022) 106:1754–61. doi: 10.1136/bjophthalmol-2021-319506, PMID: 34340976

